# Survey data on government risk communication and citizen compliance during the COVID-19 pandemic in Vietnam

**DOI:** 10.1016/j.dib.2020.106348

**Published:** 2020-09-29

**Authors:** Le Thanh Tung, Pham Tien Thanh

**Affiliations:** a Faculty of Economics and Public Management, Ho Chi Minh City Open University, Ho Chi Minh City, Vietnam; b Faculty of Business Administration, Ton Duc Thang University, Ho Chi Minh City, Vietnam

**Keywords:** COVID-19, citizen compliance, online survey, government response, government risk communication, risk perception

## Abstract

In response to the global call for data to understand the COVID-19 and thus contain its outbreak, our datasets provide COVID-19-related information in Vietnam, a limited-resource country with some achievements in the fight against this infectious disease. The survey collected various information from the respondents, including their socioeconomic characteristics, their responses on the government risk communication, their understandings of COVID-19, their risk perception of COVID-19, and their compliance with safety measures. The survey was conducted on 467 respondents living in Vietnam in the course of COVID-19 pandemic. Data was collected through an online survey conducted from March 31 to April 3, 2020 when Vietnam started the strict nationwide social distancing rules. Our dataset could serve as a reference source for similar surveys in other countries to understand the government risk communication, the public's understandings, their risk perception, and their compliance during the on-going COVID-19 and similar crises.

## Specifications Table

**Table:** 

Subject	Social Sciences; Public Health Policy
Specific subject area	Risk communication; Safety Research; Health Crisis
Type of data	Primary data Table
How data were acquired	The data were collected using structured questionnaires through an online survey.
Data format	Raw, Analyzed Dataset in Excel and Dta (STATA) extensions
Parameters for data collection	The survey was conducted on Vietnamese citizens living in both rural and urban Vietnam during the COVID-19 pandemic.
Description of data collection	The data was collected when the Vietnamese government started to initiate strict nationwide social distancing rules. This was considered a critical period as it would determine the success or failure of Vietnam in the fight against the COVID-19 pandemic.
Data source location	Region: Asia Country: Vietnam
Data accessibility	Repository name: Mendeley Data identification number: N/a Direct URL to data: http://dx.doi.org/10.17632/78r6yfbmkk.2

## Value of the Data

• The dataset is important in providing insights into government risk communication via various information and communication channels (both social and traditional media), the public's understandings, their risk perception, and their compliance with public health safety measures during the COVID-19 pandemic.
• The dataset is useful for researchers, policymakers, and public health authorities (especially those in the low-middle income countries) because the data were collected in Vietnam, a country that has been praised for its successful containment of the COVID-19 pandemic with limited resources.
• The dataset can be used to examine the relationships among these factors, especially the role of government risk communication in shaping citizen's compliance. Similar surveys can be conducted in other countries for making comparisons.
• The survey was conducted when Vietnam started to initiate nationwide social distancing rules. This was a crucial moment as it would determine Vietnam's success or failure in the fight against this infectious disease. Therefore, it is considered an additional value of our dataset.
• Any empirical analysis associated with this dataset will yield some important practices and policy implications for other limited-resource countries in their fight against the on-going COVID-19 pandemic and similar health crises. Thenceforth, it may also contribute to the literature on public risk management in the context of health and/or social crisis.

## 1 Data Description

COVID-19 has been the most challenging global crisis since World War II [1]. Many empirical works have been conducted to capture a better understanding of this fatal virus and how to contain its outbreak. The successful containment of a pandemic like COVID-19 required rapid and effective responses from the governments [2]. Government risk communication has been considered an important work to improve public awareness, which can thereby prevent this infectious disease from breaking out 9 [3], [4], [5]. Therefore, this dataset provides insightful information on government risk communications, the public's understanding, their risk perception, and their compliance with the safety measures during the COVID-19 pandemic.

The data was collected from 467 respondents using an online survey. The survey was conducted from March 31 to April 3, 2020. The dataset includes five major groups of information: (1) socioeconomic characteristics of the respondents (Table 1; Supplementary files: Part A); (2) the respondents’ self-report on the government risk communications via various information and communication channels (Table 2; Supplementary files: Part B); (3) the respondents’ understandings of COVID-19 (Table 3; Supplementary files: Part C); (4) the respondents’ perception on risks of COVID-19 (Table 4; Supplementary files: Part D); and (5) the respondents’ compliance with the public health safety measures during the pandemic (Table 5; Supplementary files: Part E). The definitions and some descriptive statistics of these information are briefly reported in Table 1, Table 2, Table 3, Table 4, Table 5. The questionnaire and codes are provided as supplementary files.

**Table 1:** Sample profiles.

Variable	Description/ Label and codes in dataset	*N*	*%*
GENDER	Gender		
	= 0 if Female	302	64.7%
	= 1 if Male	165	35.3%
AGEGROUP	Age group		
	= 1 if 15–24 years old	346	74.09%
	= 2 if 25–34 years old	75	16.06%
	= 3 if 35–49 years old	46	9.85%
LOCATION	Location		
	= 0 if Rural	174	37.3%
	= 1 if Urban	293	62.7%
EDUCATE	Education level		
	= 1 if High school and lower	28	6.0%
	= 2 if College, University	387	82.9%
	= 3 if MA/MSc	52	11.1%
INCOME	Income per capita per month		
	= 1 if <2 million VND	110	23.6%
	= 2 if ≥2 - 5 million VND	157	33.6%
	= 3 if ≥ 5 - 10 million VND	81	17.3%
	= 4 if ≥10 - 20 million VND	75	16.1%
	= 5 if ≥20 million VND	44	9.4%
**Total**		467	100.0%

*Note*: Variable column provides the variable name in the dataset.

Refer to the supplementary files (Questionnaire, and Codes and data): Part A.

**Table 2:** Information and communication channels from which the respondent have received COVID-19-related information from the government.

Variable	Description/ Label	Never receive	Rarely receive	Regularly receive	*N*
GRCZALO	Zalo	17	38	412	467
GRCSMS	SMS	18	33	416	467
GRCFACE	Facebook	70	90	307	467
GRCTIVI	Television	56	64	347	467
GRCPNEW	Printed newspaper	268	115	84	467
GRCONEW	Online newspaper	47	77	343	467
GRCRADI	Radio	312	93	62	467
GRCYUTU	YouTube	54	115	298	467

*Note*: MIN=0, MAX=2.

Codes in dataset: 0=Never receive; 1=Rarely receive; 2=Regularly receive.

The variable column provides the variable name in the dataset.

Refer to the supplementary files (Questionnaire, and Codes and data): Part B.

**Table 3:** Understandings of COVID-19.

Variable	Description/ Label	Response		*N*
		NO	YES	
KNOW1	COVID-19 was originated from Wuhan	28	439	467
KNOW2	COVID-19 is transmitted by direct contact with infected persons	86	381	467
KNOW3	There were effective vaccines or drugs for the treatment of COVID-10	462	5	467
KNOW4	Garlic could be used for containment of COVID-19	452	15	467
KNOW5	Symptoms of COVID-19 include fever, cough, sore throat, and muscle pain	30	437	467

*Note*: MIN=0, MAX=1.

Codes in dataset: 0=NO (Disagree); 1=YES (Agree).

The variable column provides the variable name in the dataset.

Refer to the supplementary files (Questionnaire, and Codes and data): Part C.

**Table 4:** Perception on risks of COVID-19.

Variable	Description/ Label	Strongly disagree	Disagree	Neutral/ No opinion	Agree	Strongly agree	*N*
RISKENVI	Risk for environment	24	55	49	143	196	467
RISKPUHE	Risk for public health	18	1	5	100	343	467
RISKSPIRI	Risk for spiritual life	6	25	64	193	179	467
RISKECON	Risk for economic life	7	3	6	118	333	467

*Note*: MIN=1, MAX=5.

Codes in dataset: 1=Strongly disagree; 2=Disagree; 3=Neutral/ No opinion; 4=Agree; 5=Strongly agree.

The variable column provides the variable name in the dataset.

Refer to the supplementary files (Questionnaire, and Codes and data): Part D.

**Table 5:** Compliance with public health safety measures.

Variable	Description/ Label	Adoption		*N*
		NO	YES	
COMPLY1	Official information seeking	156	311	467
COMPLY2	Non-essential travel minimization	25	442	467
COMPLY3	Face mask wearing	9	458	467
COMPLY4	2-meter physical distance	120	347	467
COMPLY5	Regular hand hygiene	35	432	467
COMPLY6	Healthy diet	132	335	467
COMPLY7	Physical exercising	168	299	467
COMPLY8	House hygiene	106	361	467
COMPLY9	Medical declaration	111	356	467

*Note*: MIN=0, MAX=1.

Codes in dataset: 0=NO (Not adopt), 1=YES (Adopt).

The variable column provides the variable name in the dataset.

Refer to the supplementary files (Questionnaire, and Codes and data): Part E.

## 2 Experimental Design, Materials and Methods

The survey was conducted from March 31 to April 3, 2020, when Vietnam started to initiate the strict nationwide social distancing rules to contain the outbreak of COVID-19. This was considered a critical moment as it would determine whether Vietnam would succeed in containing this infection disease. Therefore, we chose this moment to conduct the survey in order to collect information on the public's perception of government risk communication, their understandings, their risk perception, and their compliance with safety measures.

The data was collected from the Vietnamese citizens living in Vietnam pre and during the pandemic through an online survey. Prior to the official survey, a pilot test with 10 Vietnamese citizens was conducted to ensure the logical consistency, wording, meaning, and appropriateness of each question. Google form was used as the platform for hosting the questionnaire. Official survey was conducted through a public online survey using a convenient sampling method. No inducements for respondents were offered, and their anonymity was assured. A total of 471 respondents were voluntary to participate in the survey. The information collected from the online google form was automatically exported to an Excel file. Data was then cleansed using STATA. The respondents who did not complete the questionnaire or provided conflicting information were removed from the sample. After data cleansing, the final sample is 467. The dataset includes the following information:

• ***Socioeconomic characteristics***. The respondents were asked for several socioeconomic questions, including gender (2 options, male or female), age (3 options), income (5 options), education level (3 options), location (2 options, rural or urban).
• ***Government risk communication***. 8 popular information and communication channels that the government use to communicate risk to the public were selected to survey, including Zalo (a Vietnam's social network), SMS, Facebook, TV, online newspapers, printed newspapers, radio, and YouTube. For each channel, the respondents self-reported on whether they “never receive”, “rarely receive”, or “regular receive” COVID-19-related information from the government.
• ***Understanding.*** 5 questions were asked to test for the respondents’ understanding of COVID-19. The respondent self-reported on whether they “agree” or “disagree” with the 5 corresponding statements. In other words, the respondents would self-report “YES” if they thought the statement is correct, and “NO” otherwise.
• ***Risk perception****.* The respondents were asked for their perception of 4 risk problems of COVID-19. These questions were measured using 5-point Likert-type scales, ranging from 1 (strongly disagree) to 5 (strongly agree). Therefore, the respondent self-reported on whether they “strongly disagree”, “disagree”, “neutral or no opinion”, “agree” and “strongly agree” with 4 risk statements.
• ***Compliance with public health safety measure****.* Table 5 provides information on the respondent's compliance with safety measures. 9 safety measures were selected based on the important prevention and control measures recommended by the World Health Organization and the Ministry of Health of Vietnam. The respondent self-reported whether they adopted any safety measure or not. In other words, the respondents would self-report “YES” if they adopted, and “NO” otherwise.

Further studies could re-use or develop our datasets to capture better understanding or to make comparisons across countries during the COVID-19 pandemic.

## Ethics Statement

• *Authorship of the paper*: We confirm that the data article has been read and approved by all named authors and that there are no other persons who satisfied the criteria for authorship but are not listed. We further confirm that the order of authors listed in the manuscript has been approved by all of us. The contribution of each author is as follows: (1) **Le Thanh Tung**: Conceptualization; Methodology; Writing- Original draft preparation; Data collections ; (2) **Pham Tien Thanh**: Data collection and cleansing; Data Curation; Writing- Reviewing and Editing.
• *Originality and plagiarism:* We ensure that we have written entirely original works. The work and/or words of others have been appropriately cited or quoted.
• *Multiple, redundant, or concurrent publication*: We declare that this data article is original, has not been published before, and is not currently being considered for publication elsewhere.
• *Acknowledgement of sources*: Proper acknowledgement of the work of others was given.
• *Fundamental errors in published works*: When we discover a significant error or inaccuracy in our own published work, we will be responsible to promptly notify the journal editor or publisher and cooperate with the editor to retract or correct the paper.
• *Informed consent*: We confirm that the participants were asked for individual consent before the interview, and that the informed consent has been obtained from all the participants. All procedures performed in this study are in accordance with the ethical standards of the institutional and/or national research committee and with the 1964 Helsinki declaration and its later amendments or comparable ethical standards.

## Funding

None.

## Declaration of Competing Interest

The authors declare that they have no known competing financial interests or personal relationships which have, or could be perceived to have, influenced the work reported in this article.

## Appendix Supplementary materials

Image, application 1

Image, application 2
