# Supplementary material for: Survey data on government risk communication and citizen compliance during the COVID-19 pandemic in Vietnam
Source: Data Brief. 2020 Sep 29;33:106348. doi: 10.1016/j.dib.2020.106348 (PMC7524451; doi:10.1016/j.dib.2020.106348)
Supplement: Supplementary file 1 [file mmc1.docx]

**ID: ……….**

Our research group would like to conduct a survey on *"Government risk communication and citizen compliance during the COVID-19 pandemic in Vietnam”*. Please take a few minutes to give us your valuable feedback on each question. We guarantee that the information will be used for research purpose only.

Thank you very much for your time and your support.

# PART A. GENERAL INFORMATION

**Q.A1: Your gender?**

🞏 Female 🞏 Male

**Q.A2: Your age?**

🞏 15–24 years old

🞏 25–34 years old

🞏 35–49 years old

**Q.A3: Your location?**

🞏 Rural 🞏 Urban

**Q.A4: Your education?**

🞏 High school and lower

🞏 College, University

🞏MA/MSc

**Q.A5: Your income per capita per month? (Including private transfer from family members or relatives)**

🞏 <2 million VND

🞏 ≥2 - 5 million VND

🞏 ≥ 5 - 10 million VND

🞏 ≥10 - 20 million VND

🞏 ≥20 million VND

# PART B. RISK COMMUNICATION

**From which information and communication channels have you received COVID-19 information from the government?**

|  | Never receive | Rarely receive | Regularly receive |
| --- | --- | --- | --- |
| **Q.B1: Zalo** | 🞏 |  | 🞏 |
| **Q.B2: SMS** | 🞏 |  | 🞏 |
| **Q.B3: Facebook** | 🞏 |  | 🞏 |
| **Q.B4: Television** | 🞏 |  | 🞏 |
| **Q.B5: Printed newspaper** | 🞏 |  | 🞏 |
| **Q.B6: Online newspaper** | 🞏 |  | 🞏 |
| **Q.B7: Radio** | 🞏 |  | 🞏 |
| **Q.B8: YouTube** | 🞏 |  | 🞏 |

# PART C: KNOWLEDGE

**Do you agree with the following statements?**

|  | NO (Disagree) | YES (Agree) |
| --- | --- | --- |
| **Q.C1: COVID-19 was originated from Wuhan** | 🞏 | 🞏 |
| **Q.C2: COVID-19 is transmitted by direct contact with infected persons** | 🞏 | 🞏 |
| **Q.C3: There were affective vaccines or drugs for the treatment of COVID-10** | 🞏 | 🞏 |
| **Q.C4: Garlic could be used for containment of COVID-19** | 🞏 | 🞏 |
| **Q.C5: Symptoms of COVID-19 include fever, cough, sore throat and muscle pain** | 🞏 | 🞏 |

# PART D: RISK PERCEPTION

**Please indicate the degree to which you agree or disagree with each of the following statements.**

|  | Strongly disagree | Disagree | Neutral / No opinion | Agree | Strongly agree |
| --- | --- | --- | --- | --- | --- |
| **Q.D1: The pandemic is a risk for environment** | 🞏 | 🞏 | 🞏 | 🞏 | 🞏 |
| **Q.D2: The pandemic is a risk for public health** | 🞏 | 🞏 | 🞏 | 🞏 | 🞏 |
| **Q.D3: The pandemic is a risk for spiritual life** | 🞏 | 🞏 | 🞏 | 🞏 | 🞏 |
| **Q.D4: The pandemic is a risk for economic life** | 🞏 | 🞏 | 🞏 | 🞏 | 🞏 |

# PART V: COMPLIANCE WITH SAFETY MEASURES

**Have you adopted the following safety measures to protect your-self and your family against the COVID-19 pandemic?**

|  | NO (Not adopt) | YES (Adopt) |
| --- | --- | --- |
| **Q.E1: Official information seeking** | 🞏 | 🞏 |
| **Q.E2: Non-essential travel minimization** | 🞏 | 🞏 |
| **Q.E3: Face mask wearing** | 🞏 | 🞏 |
| **Q.E4: 2-meter physical distance** | 🞏 | 🞏 |
| **Q.E5: Regular hand hygiene** | 🞏 | 🞏 |
| **Q.E6: Healthy diet** | 🞏 | 🞏 |
| **Q.E7: Physical exercising** | 🞏 | 🞏 |
| **Q.E8: House hygiene** | 🞏 | 🞏 |
| **Q.E9: Medical declaration** | 🞏 | 🞏 |
